# Laminin-1 Peptides Conjugated to Fibrin Hydrogels Promote Salivary Gland Regeneration in Irradiated Mouse Submandibular Glands

**DOI:** 10.3389/fbioe.2021.729180

**Published:** 2021-09-24

**Authors:** Kihoon Nam, Harim T. dos Santos, Frank Maslow, Bryan G. Trump, Pedro Lei, Stelios T. Andreadis, Olga J. Baker

**Affiliations:** ^1^ Bond Life Sciences Center, University of Missouri, Columbia, MO, United States; ^2^ Department of Otolaryngology-Head and Neck Surgery, School of Medicine, University of Missouri, Columbia, MO, United States; ^3^ School of Dentistry, University of Utah, Salt Lake City, UT, United States; ^4^ Department of Chemical and Biological Engineering, University at Buffalo, The State University of New York, Buffalo, NY, United States; ^5^ Department of Biomedical Engineering, University at Buffalo, The State University of New York, Buffalo, NY, United States; ^6^ Center of Bioinformatics and Life Sciences, University at Buffalo, The State University of New York, Buffalo, NY, United States; ^7^ Center of Cell, Gene and Tissue Engineering, University at Buffalo, The State University of New York, Buffalo, NY, United States; ^8^ Department of Biochemistry, University of Missouri, Columbia, MO, United States

**Keywords:** biomaterial, hydrogel, regeneration, tissue engineering, saliva, irradiated salivary glands

## Abstract

Previous studies demonstrated that salivary gland morphogenesis and differentiation are enhanced by modification of fibrin hydrogels chemically conjugated to Laminin-1 peptides. Specifically, Laminin-1 peptides (A99: CGGALRGDN-amide and YIGSR: CGGADPGYIGSRGAA-amide) chemically conjugated to fibrin promoted formation of newly organized salivary epithelium both *in vitro* (*e.g.,* using organoids) and *in vivo* (*e.g.,* in a wounded mouse model). While these studies were successful, the model’s usefulness for inducing regenerative patterns after radiation therapy remains unknown. Therefore, the goal of the current study was to determine whether transdermal injection with the Laminin-1 peptides A99 and YIGSR chemically conjugated to fibrin hydrogels promotes tissue regeneration in irradiated salivary glands. Results indicate that A99 and YIGSR chemically conjugated to fibrin hydrogels promote formation of functional salivary tissue when transdermally injected to irradiated salivary glands. In contrast, when left untreated, irradiated salivary glands display a loss in structure and functionality. Together, these studies indicate that fibrin hydrogel-based implantable scaffolds containing Laminin-1 peptides promote secretory function of irradiated salivary glands.

## Introduction

According to the American Cancer Society, each year more than 80,000 people develop head and neck cancer in the United States (Siegel et al., 2021). A first-line treatment for head and neck cancer is radiation therapy (Sroussi et al., 2017), but ionizing radiation typically leads to chronic oral complications such as xerostomia (i.e., hyposalivation) (Chambers et al., 2004; Grundmann et al., 2010; Jensen et al., 2010; Pinna et al., 2015; Sroussi et al., 2017; Jensen et al., 2019; Haderlein et al., 2020; Jasmer et al., 2020). This condition contributes to oral microbial infections and impairs activities of daily life such as speaking, chewing, and swallowing (Lovelace et al., 2014; Brook, 2021). Existing treatments for hyposalivation are limited to the use of muscarinic receptor agonists (e.g., cevimeline and pilocarpine) (Braga et al., 2009; Turner, 2016) that induce saliva secretion from the few remaining acinar cells as well as use of saliva substitutes (Silvestre et al., 2009; Rocchi and Emmerson, 2020); however, these therapies target surface-level symptoms and provide only temporary relief (Jaguar et al., 2017; Jensen et al., 2019; Lung et al., 2021). Therefore, development of alternative treatments to restore salivary gland secretory function is critical. Several experimental therapies including the use of stem cells (Nanduri et al., 2011; Nanduri et al., 2013; Pringle et al., 2013; Mitroulia et al., 2019; Su et al., 2020), embryonic organ culture (Ogawa et al., 2013; Ogawa and Tsuji, 2015; Ikeda et al., 2019), organ bioprinting (Ferreira et al., 2016; Adine et al., 2018), cell sheets (Nam et al., 2019a; dos Santos et al., 2020), gene therapy (Zheng et al., 2011; Baum et al., 2012; Arany et al., 2013) and bioengineered scaffolds (Peters et al., 2014; Foraida et al., 2017; Patil and Nanduri, 2017; Nam et al., 2019b) have offered the promise of more advanced solutions as detailed below.

Regarding stem cells/progenitors, previous studies showed that c-Kit^+^ cells, which normally are found in very low numbers within salivary gland specimens (Nanduri et al., 2011; Nanduri et al., 2013) can be expanded *ex vivo* for restoring salivary gland function; however, further characterization (e.g., how they incorporate into host tissue as well as long term secondary effects such as tumorigenesis and survival rates) must be determined before translating this approach into humans. Another technology involves the use of embryonic organ culture transplantation, where embryonic salivary cells grown in culture can be transplanted *in vivo* (Ogawa et al., 2013); nonetheless, a diminished gland size and an absence of studies showing long-term outcomes following treatment significantly decrease the utility of this model for translational applications. Bioprinting strategies have shown the possibility of assembling glandular compartments (e.g., acinar/ductal epithelial, myoepithelial, endothelial, and neuronal) into salivary gland organotypic cultures; however, this technology does not mimic the salivary gland native architecture (e.g., cell polarity and organization (Ferreira et al., 2016; Adine et al., 2018)). Cell sheets made of salivary gland cells have demonstrated positive results, as they promote cell differentiation and tissue integrity in wounded mouse submandibular gland (SMG) models, yet the main challenge facing this technology is the need to standardize cell composition within the sheets and thereby achieve greater reproducibility (Nam et al., 2019a; dos Santos et al., 2020). Regarding scaffolds other than the Fibrin Hydrogels (FH), various biomaterials (Aframian et al., 2000; Sun et al., 2006; Cantara et al., 2012; Soscia et al., 2013; Hsiao and Yang, 2015; Yang and Hsiao, 2015) have been shown to promote cell growth and attachment but the degree of structural organization, as demonstrated by hollow multi-lumen formation, cell polarity and functionality, has been modest. Likewise, studies have shown that human cells grown on a hyaluronic acid-based scaffold and transplanted into a wounded mouse parotid gland lead to improved secretory function (Pradhan-Bhatt et al., 2014); nevertheless, these results included neither monitoring for degradation of the scaffold nor evidence of new tissue formation, thus raising concerns with the stability of the biomaterial and capacity for regeneration, respectively. Together, these technologies offer the potential for more advanced solutions to hyposalivation due to head and neck radiation therapy but have yet to truly deliver.

In response to these needs and challenges, we developed FH with conjugated Laminin-1 peptides (L_1p_) A99 and YIGSR that were used successfully to repair salivary gland tissue in a wounded SMG mouse model (Nam et al., 2017a; Nam et al., 2017b; Nam et al., 2019b). To apply these results to a more translational setting, the goal of the current study is to determine whether transdermal injection with the L_1p_ A99 and YIGSR chemically conjugated to FH can promote secretory function in irradiated salivary glands.

## Materials and Methods

### Materials

Lyophilized human fibrinogen, tris base, ethylenediaminetetraacetic acid (EDTA), pilocarpine, isoproterenol, goat serum, hydrochloric acid, hematoxylin, eosin Y solution, Tween® 20, calcium chloride (CaCl_2_) and ε-aminocaproic acid (εACA) were purchased from MilliporeSigma (Burlington, MA). Rabbit anti-zonula occludens 1 (ZO-1) antibody, rabbit anti-induced nitric oxide synthase (iNOS) antibody, Alexa Fluor 488 conjugated anti-rabbit IgG secondary antibody, Alexa Fluor 568 conjugated anti-rabbit IgG secondary antibody and Alexa Fluor 568 conjugated anti-mouse IgG secondary antibody were purchased from Invitrogen (Carlsbad, CA). Rabbit anti-transmembrane Protein 16A (TMEM16A) antibody and mouse anti-intercellular adhesion molecule (ICAM-1) antibody were purchased from Abcam (Cambridge, MA). Rabbit anti-vascular cell adhesion molecule 1 (VCAM-1) antibody and rabbit Arginase-1 (Arg-1) antibody were purchased from Cell Signaling Technology (Danvers, MA). Mouse anti-Na^+^/K^+^-ATPase antibody was purchased from Santa Cruz Biotechnology (Dallas, TX). Mouse anti-E-cadherin antibody was purchased from BD Biosciences (San Jose, CA). Phosphate buffered saline (PBS), DyLight™ 680 NHS-ester, 4′,6-diamidino-2-phenylindole (DAPI), Triton X-100, sodium citrate, xylene and ethanol were purchased from Thermo Fisher Scientific (Waltham, MA). Ketamine and xylazine were purchased from VetOne (Boise, ID). Insulin syringes (28G) were purchased from BD (Franklin Lakes, NJ). Peptides were synthesized by University of Utah DNA/Peptide synthesis core facility, as previously described (Nam et al., 2016; Nam et al., 2017a; Nam et al., 2017b).

### Animals

Female 6-week-old C57BL/6J mice weighing ∼17–20 g were purchased from Jackson Laboratory (Bar Harbor, ME). Power analysis was performed to determine mouse numbers using G^*^Power 3.1.9.7 software (Heinrich-Heine-Universität Düsseldorf, Düsseldorf, Germany; http://www.gpower.hhu.de/). All calculations were conducted using a significance level of 0.05 with 95% power. Then, 105 mice were randomly distributed into three groups to receive the following treatments: non-irradiated (40 mice), irradiated without L_1p_-FH injection (40 mice), and irradiated while also receiving the L_1p_-FH injection (25 mice), comprising treatment groups 1–3, respectively. All animal usage, anesthesia and surgeries were conducted with the approval of the University of Utah Institutional Animal Care and Use Committee (IACUC) in compliance with the ARRIVE guidelines.

### Radiation Treatment

**FIGURE 1:**
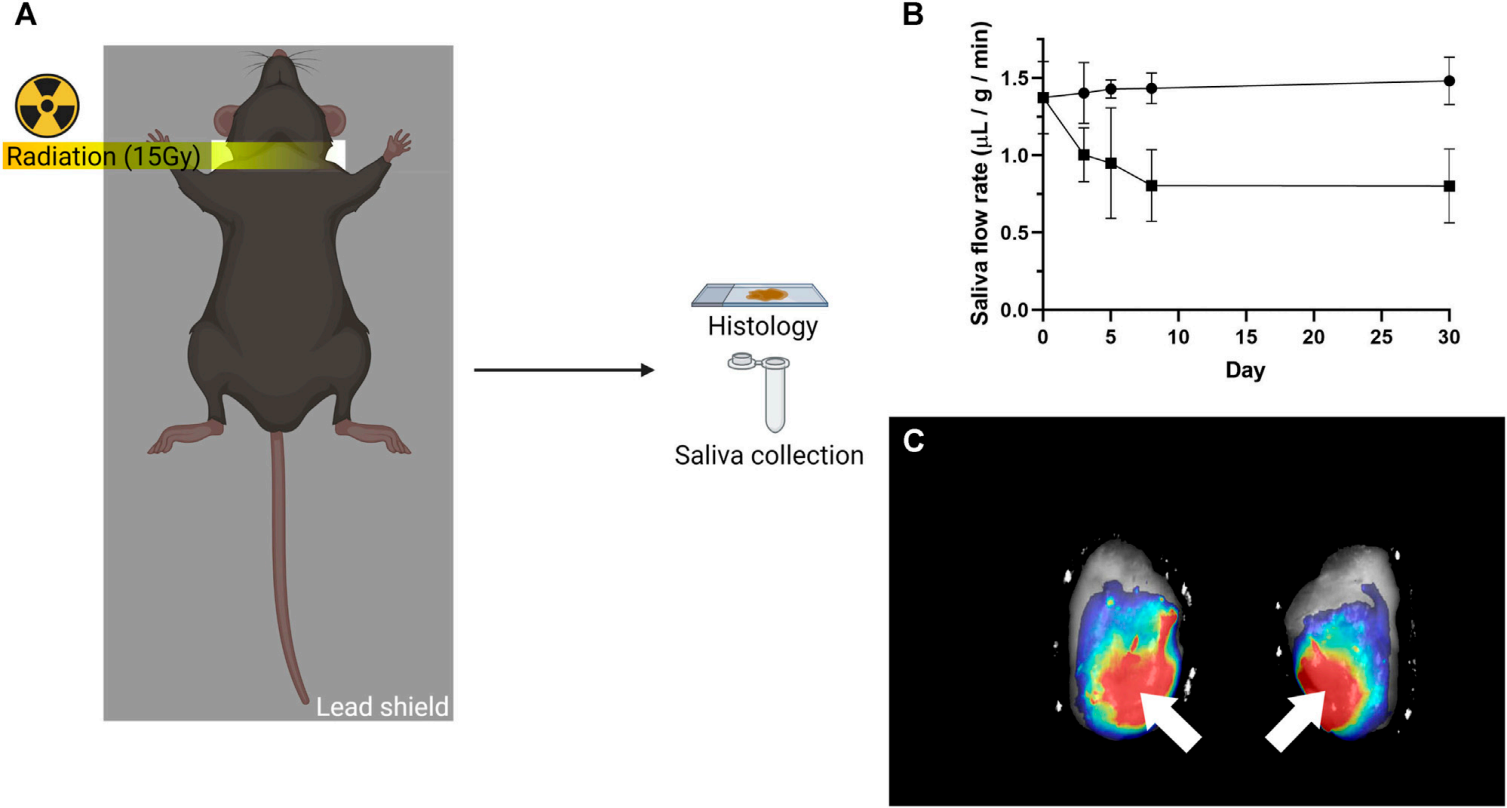
Radiation treatment and local L_1P_-FH delivery used in this study. **(A)** Mice received a single 15 Gy radiation dose with a customized lead shield having a 1 cm slit aligned to the mouse’s neck. **(B)** Radiation treatment caused saliva flow rates to be significantly reduced. The symbol (•) indicates non-irradiated group, while the symbol (■) indicates irradiated group. **(C)** DyLight 680 conjugated L_1P_-FH were successfully delivered to the mouse submandibular glands when applied via transdermal injection. White arrows indicate the site of L_1p_-FH injection.

Salivary gland tissue damage is a late degenerative response observed after radiation therapy (Wu and Leung, 2019; Jasmer et al., 2020). To confirm L_1p_-FH regenerative effects in a more clinically relevant animal model, a widely accepted head and neck irradiated mouse model was used for this study (Deasy et al., 2010; Varghese et al., 2018). Briefly, mice were anesthetized with ketamine (100 mg/kg) and xylazine (5 mg/kg) solution administered intraperitoneally with the head and neck area positioned over the 1 cm slit of a customized lead shield, thereby protecting other areas of the body from radiation. SMGs then received a single 15 Gy radiation dose using a JL Shepherd ^137^Cs irradiator (Figure 1A). Animals were allowed to recover for 3 days and received hydrogel treatment soon after, as detailed below.

### Hydrogel Preparation

Peptides and DyLight 680 conjugated fibrinogen were prepared, as previously described (Nam et al., 2017a; Nam et al., 2017b). Briefly, two Laminin-1 peptides (A99 and YIGSR) were synthesized on a peptide synthesizer. Peptides were then conjugated to the fibrinogen using sulfo-LC-SPDP and cysteine residue in peptides. In addition, fibrinogen was chemically labeled with a fluorescent dye through NHS ester of DyLight 680. Finally, laminin-1 peptide conjugated fibrinogens and DyLight 680 labeled fibrinogen were dialyzed against ultrapure water, lyophilized, and stored at −80°C until use. L_1p_-FH were prepared similar to previous studies (Nam et al., 2017a; Nam et al., 2017b) except for the use of exogenous thrombin (thereby preventing rapid polymerization inside the syringe) as follows: YIGSR-conjugated fibrinogen (1.2 mg/ml), A99-conjugated fibrinogen (1.2 mg/ml), DyLight 680 conjugated fibrinogen (0.1 mg/ml), CaCl_2_ (2.5 mM) and εACA (2 mg/ml) were mixed in a tris buffered saline (TBS) solution. Polymerization of L_1p_-FH was confirmed from fluorescence in the SMG of randomly selected mice (Figure 1C).

### Transdermal Injection

C57BL/6J mice were anesthetized with 3% isoflurane using an oxygen flow rate set at 2.0 L/min, and 10 μL of freshly mixed L_1p_-FH solution was transdermally injected using insulin syringe (G 28) to irradiated mouse SMGs at post-radiation day 3. L_1p_-FH effects were studied at days 8 and 30. Using thrombin prior transdermal injection causes rapid polymerization of L_1p_-FH which clogs the needle. To overcome this issue, the mixture was applied in a liquid form using endogenous thrombin for internal polymerization. To confirm scaffold implantation *in vivo*, FH was labeled with DyLight 680 and quantified within dissected glands using a Bio-Rad Chemi-Doc™ MP imaging system (Figure 1C).

### Hematoxylin and Eosin and Masson’s Trichrome Stain

SMGs were fixed in 10% formalin at room temperature overnight, dehydrated in 70% ethanol solution, embedded in paraffin wax and cut into 3 μm sections. Sections were then deparaffinized with xylene and rehydrated with serial ethanol solutions (100%, 95% 80, 70 and 50%, v/v) and distilled water. For hematoxylin and eosin staining, the rehydrated sections were stained with hematoxylin for 5 min, washed with distilled water for 5 min, tap water for 5 min and distilled water for 2 min. Next, slides were stained with eosin for 30 s, washed with tap water for 5 min and distilled water for 2 min. Finally, hematoxylin and eosin stained gland sections were dehydrated with 95 and 100% ethanol (v/v), cleared in xylene and mounted with a xylene-based mounting medium. As for Masson’s trichrome staining, the rehydrated sections were re-fixed in Bouin’s solution at 60°C for 1 h then washed with running tap water for 10 min and distilled water for 5 min. Next, sections were stained with Weigert’s iron hematoxylin solution for 10 min then washed with running warm tap water for 10 min and distilled water for 5 min. For cytoplasm staining, sections were incubated with Biebrich scarlet acid fuchsine solution for 5 min and washed three times with distilled water for 2 min. Regarding collagen staining, sections were incubated in phosphotungstic/phosphomolybdic acid for 15 min, stained with aniline blue solution for 5 min and washed three times with distilled water for 2 min. Stained sections were then differentiated in 1% acetic acid solution for 1 min and washed two times with distilled water for 2 min. Finally, Masson’s trichrome stained sections were dehydrated with serial ethanol solutions (95 and 100%), cleared in xylene and mounted with a xylene-based mounting medium. Finally, the samples were analyzed using a Leica DMI6000B (Leica Microsystems, Wetzlar, Germany) to determine tissue morphology.

### Confocal Analysis

For antigen retrieval, the rehydrated and fixed tissue sections were incubated in Tris-EDTA buffer [10 mM Tris, 1 mM EDTA, 0.05% (v/v) Tween® 20, pH 9.0] for ZO-1 and E-cadherin or with sodium citrate buffer [10 mM sodium citrate, 0.05% (v/v) Tween® 20, pH 6.0] for TMEM16A, Na^+^/K^+^-ATPase, iNOS, Arg-1, VCAM-1 and ICAM-1 at 95°C for 30 min. Next, samples were permeabilized with 0.1% (v/v) triton X-100 in PBS at room temperature for 45 min. Specimens were then blocked in 5% (v/v) goat serum in PBS for 1 h at room temperature and incubated at 4°C with the following primary antibodies overnight: rabbit anti-ZO-1, mouse anti-E-cadherin, rabbit anti-TMEM16A, mouse anti-Na^+^/K^+^-ATPase, rabbit anti-VCAM-1 or mouse anti-ICAM-1. At that time, sections were incubated with anti-rabbit Alexa Fluor 488 and anti-mouse Alexa Fluor 568 secondary antibodies in 5% goat serum at room temperature for 1 h followed by 300 nM DAPI staining at room temperature for 5 min. For M1 and M2 marker staining, specimens were blocked in 3% (w/v) bovine serum albumin (BSA) in PBS for 1 h at room temperature and incubated with primary antibodies (rabbit anti-iNOS or rabbit anti-Arg-1) at 37°C for 1 h. Then, sections were incubated with anti-rabbit Alexa Fluor 568 in 3% BSA at room temperature for 1 h followed by 300 nM DAPI staining at room temperature for 5 min. Finally, specimens were analyzed using a STELLARIS Confocal Microscope (Leica Microsystems, Wetzlar, Germany).

### Macrophage Ratio

M1 and M2 macrophage cells were determined using ImageJ. Specifically, the color threshold was set to isolate the colocalized signal of nuclei and M1 (Figure 4, white arrows)/M2 (Figure 4, red arrows) positive cells, which were counted and normalized by area. Statistical significance was assessed using one-way ANOVA (*p < 0.01) and Dunnett’s post-hoc test for multiple comparisons to group 2 (irradiated with no L_1p_-FH injection at day 30).

### Saliva Flow Rate Measurements

Mice were anesthetized with ketamine (100 mg/kg) and xylazine (5 mg/kg) followed by intraperitoneal injection with pilocarpine (25 mg/kg) and isoproterenol (0.5 mg/kg). Then, whole saliva was collected using a micropipette for 5 min and flow rate was calculated using the following formula:

$$\text{Saliva flow rate}=\;\frac{\text{Stimulated saliva }\left(\text{µL}\right)}{\text{Body weight of mouse }\left(\text{g}\right)\text{ x collection time }\left(5\text{min}\right)}$$

### Statistical Analysis

Experimental data were analyzed using one-way ANOVA and Dunnett’s post hoc test for multiple comparisons to the non-irradiated group 1 at day 30. All values represent means ± SD (*n* = 5), where *p* values <0.01 were considered statistically significant. Finally, these calculations were performed using GraphPad Prism 6.

## Results

### A Head and Neck Irradiated Mouse Model was Achieved

To investigate whether L_1p_-FH could restore irradiated SMG structure and function, C57BL/6J mice were subjected to a single radiation treatment as described in Materials and Methods (Figure 1A). Mice treated with a single 15 Gy radiation dose displayed a significant reduction in saliva flow rates as compared to non-irradiated controls (i.e., from 1.43 to 0.80 μL/g/min, *n* = 5, *p* < 0.01) in the first 8 days and remained steady thereafter until day 30 (Figure 1B). These results demonstrated that the radiation dose utilized here caused significant loss of salivary secretory function and can thus be used as a head and neck irradiated preclinical model, consistent with previous studies (Lombaert et al., 2008; Varghese et al., 2018; Weng et al., 2018).

### L_1p_-FH was Successfully Implanted in Irradiated Mouse Submandibular Glands

Our previous studies showed the biocompatibility of L_1p_-FH with host tissue when surgically implanted in a wounded mouse model (Nam et al., 2017a; Nam et al., 2017b). To avoid an open wound surgery, we attempted to deliver the L_1p_-FH to irradiated mouse SMG via transdermal injection as described in Materials and Methods. For these experiments, we used a fluorescently labeled hydrogel using DyLight 680 and successfully implanted L_1p_-FH in irradiated mouse SMG *via* transdermal injection (Figure 1C, white arrows).

### L_1p_-FH Preserved Epithelial Integrity After Radiation Treatment

**FIGURE 2:**
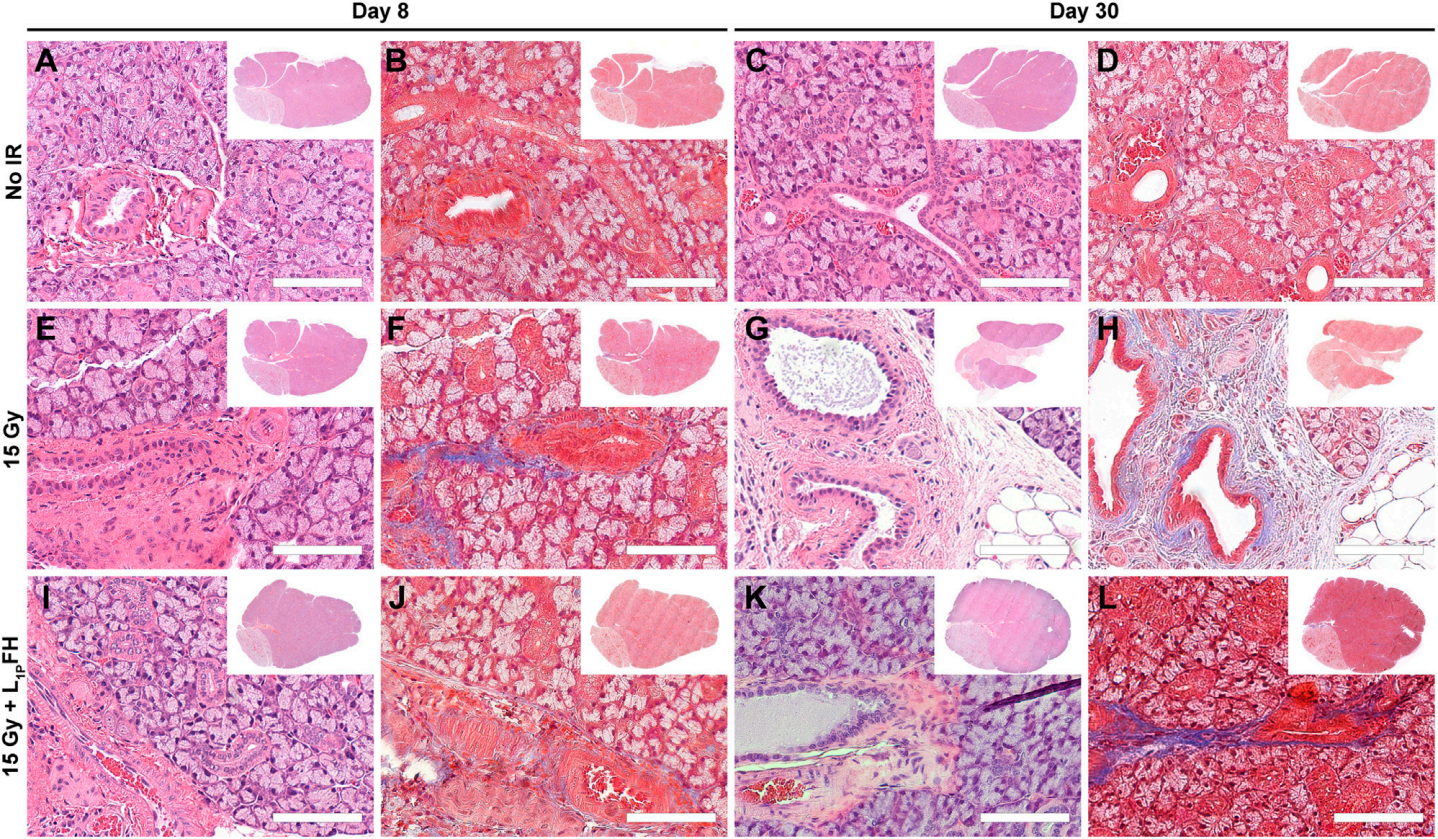
Treatment with L_1p_-FH preserves epithelial integrity when applied after radiation treatment. Hematoxylin and eosin **(A,C,E,G,I,K)** as well Masson’s trichrome **(B,D,F,H,J,L)** staining of mouse submandibular glands from group 1 [non-irradiated, **(A–D)**], group 2 [irradiated without L_1p_-FH injection, **(E–H)**] and group 3 [irradiated with L_1p_-FH injection, **(I–L)**] were performed and tissue morphology was analyzed using a Leica DMI6000B. Scale bars represent 100 µm. Representative image from a total of five mice per group.

Our previous studies showed that L_1p_-FH promoted tissue repair in a wounded SMG mouse model (Nam et al., 2017a; Nam et al., 2017b; Nam et al., 2019b). To determine whether these effects occur in the head and neck irradiated mouse model, we randomly distributed mice in three groups and applied this scaffold as follows: non-irradiated, irradiated without L_1p_-FH injection and irradiated that received the L_1p_-FH injection, comprising treatment groups 1–3, respectively (see Material and Methods section). As shown in Figure 2, group 1 (non-irradiated glands) displayed intact lobules where the parenchyma was separated by areas of thin connective tissue at days 8 (Figures 2A,B) and 30 (Figures 2C,D). As for cytologic features, serous acini cells showed a typical pyramidal shape with basophilic cytoplasm and basal nuclei. In contrast, mucous cells showed a pale cytoplasm with flat basilar nuclei, intercalated ducts were lined by cuboidal and/or flat cells, striated ducts showed cuboidal to low columnar cells and granular convoluted ducts were lined by tall columnar cells containing intracytoplasmic eosinophilic granules. Together, these features indicate that the non-irradiated glands in group 1 showed the morphology of a healthy epithelium. In contrast, group 2 (irradiated with no L_1p_-FH injection) demonstrated glandular parenchyma separated by thicker connective tissue strands, ductal areas with ectasia, intraluminal depositions and increased presence of fibrosis when compared to controls (Figures 2E,F). Furthermore, tissue damage was even more severe at day 30 (Figures 2G,H), where SMG showed an extensive disruption of the lobular architecture as indicated by the replacement of acini and ducts with sheets of vacuolated cells, adipocytes and fibrosis. Together, these results indicated that irradiated glands with no L_1p_-FH injection (group 2) dramatically lost epithelial integrity. Remarkably, mice in group 3 (irradiated with L_1p_-FH injection) recovered many of the features of healthy glands. For instance, we observed the presence of serous acinar units with organized ductal structures surrounded by thin connective tissue strands similar to the non-irradiated group 1 at both days 8 (Figures 2I,J) and 30 (Figures 2K,L). These changes indicate that group 3 (irradiated glands treated with L_1p_-FH) had a morphology consistent with a healthy salivary gland epithelium and results in this section indicate that L_1p_-FH is a suitable scaffold for promoting epithelial integrity in irradiated SMG.

### L_1p_-FH Maintained Epithelial Polarity and Preserved Ion Transporter Expression

**FIGURE 3:**
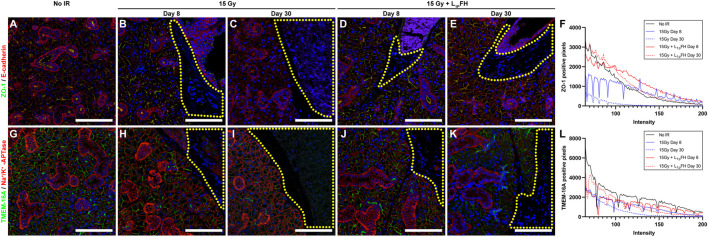
Treatment with L_1p_-FH maintains epithelial polarity and functional marker expression. Salivary structural and functional marker organization was analyzed using confocal microscopy with specific antibodies against ZO-1 [green; **(A–E)**], E-cadherin [red; **(A–E)**], TMEM16A [green; **(G–K)**], Na^+^/K^+^-ATPase [red; **(G–K)**], and DAPI (blue; everywhere). Scale bars represent 100 µm. Yellow-dotted areas indicate fibroblast-like areas. Representative image from a total of five mice per group. ZO-1 **(F)** and TMEM-16A **(L)** positive pixels were analyzed using ImageJ.

To determine whether L_1p_-FH maintained epithelial polarity in an irradiated mouse model, we stained the SMG sections with the apical tight junction marker ZO-1 and basolateral marker E-cadherin. As shown in Figure 3A, group 1 (non-irradiated glands) displayed apical ZO-1 (green) and basolateral E-cadherin (red) after 30 days. However, in group 2 (irradiated glands with no L_1p_-FH injection), a mild residual ZO-1 signal was detected at day 8 (Figures 3B,F, blue solid line), and a weaker ZO-1 signal was expressed at day 30 (Figure 3F, blue dotted line), together with ZO-1 disorganization (Figure 3C), thereby indicating loss of epithelial polarity. In contrast, group 3 (irradiated glands treated with L_1p_-FH) showed apical ZO-1 and basolateral E-cadherin signals both at days 8 (Figure 3D) and 30 (Figure 3E), indicating that the scaffold treatment helps to maintain epithelial polarity (Figure 3F, red line and red dotted line). Regarding the presence of functional markers, group 1 (non-irradiated SMG) showed apical TMEM16A (Figure 3G, green) and basolateral Na^+^/K^+^-ATPase localization (Figure 3G, red) at day 30, consistent with a healthy salivary epithelium. In contrast, group 2 (irradiated glands with no L_1p_-FH injection) showed a moderate TMEM16A signal (Figure 3L, blue solid line) at day 8 (Figure 3H, green) and weaker TMEM16A signal (Figure 3L, blue dotted line) at day 30 (Figure 3I, green). Interestingly, group 3 (irradiated glands treated with L_1p_-FH) expressed strong apical TMEM16 (Figures 3J,K, green; Figure 3L, red line and red dotted line) and basolateral Na^+^/K^+^-ATPase similar to non-irradiated glands, thus suggesting that L_1P_-FH treatment helps to maintain epithelial polarity and preserve ion transport expression, both of which are critical for saliva secretion.

### L_1p_-FH Promoted Macrophage Polarization

**FIGURE 4:**
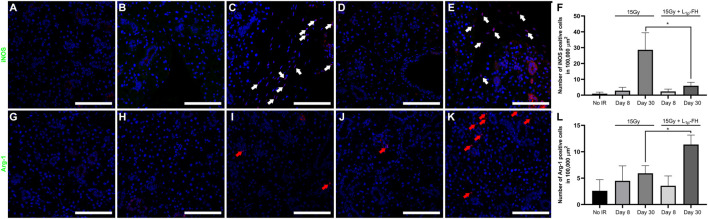
L_1p_-FH promotes macrophage polarization. Macrophage marker expression was analyzed using confocal microscopy with specific antibodies against iNOS **(A–F)**, Arg-1 **(G–L)**, and DAPI (blue; everywhere). Scale bars represent 100 µm. White and red arrows indicate iNOS or Arg-1 positive cells, respectively. Representative image from a total of five mice per group. iNOS **(F)** and Arg-1 **(L)** positive cells were analyzed using ImageJ and GraphPad Prism 6. Data represent the means ± SD of *n* = 5 mice per condition with statistical significance assessed using one-way ANOVA (*p < 0.01) and Dunnett’s post-hoc test for multiple comparisons to group 2 (irradiated with no L_1p_-FH injection at day 30).

Our previous studies indicated that treatment with L_1p_-FH promoted macrophage polarization in a wounded SMG female mouse model (Brown et al., 2020). To determine whether similar effects occur in an irradiated mouse model, we identified the presence of M1 and M2 subtypes within the SMG using macrophage-specific antibodies (i.e., iNOS and Arg-1, corresponding to M1 and M2, respectively). As shown in Figures 4A,F, group 1 (non-irradiated glands) expressed iNOS-positive cells with approximately 0.94 macrophages per 100,000 µm (Sroussi et al., 2017). In contrast, group 2 (irradiated glands with no L_1p_-FH injection) showed a significant increase in M1 macrophages (approximately 28.65 iNOS-positive cells) at day 30 (Figures 4C,F). Notably, group 3 (irradiated glands treated with L_1p_-FH) showed a significant decrease of M1 macrophages (approximately 5.92 iNOS-positive cells) at day 30 (Figures 4E,F) compared to group 2. Regarding the presence of M2 markers, group 2 (irradiated glands with no L_1p_-FH injection) expressed Arg-1-positive cells with approximately 5.92 macrophages at day 30 (Figures 2I,L), which is not a significant difference from group 1 (Figures 4G,L, 2.60 macrophages). Interestingly, group 3 (irradiated glands treated with L_1p_-FH) expressed a significant increase of Arg-1-positive cells at day 30 (approximately 11.37 macrophages, Figure 4K,L). Together, these results indicate that L_1p_-FH causes a decrease in M1 macrophages together with an increase in M2 macrophages in SMG following radiation treatment.

### L_1p_-FH Increased Saliva Secretion After Radiation Treatment

**FIGURE 5:**
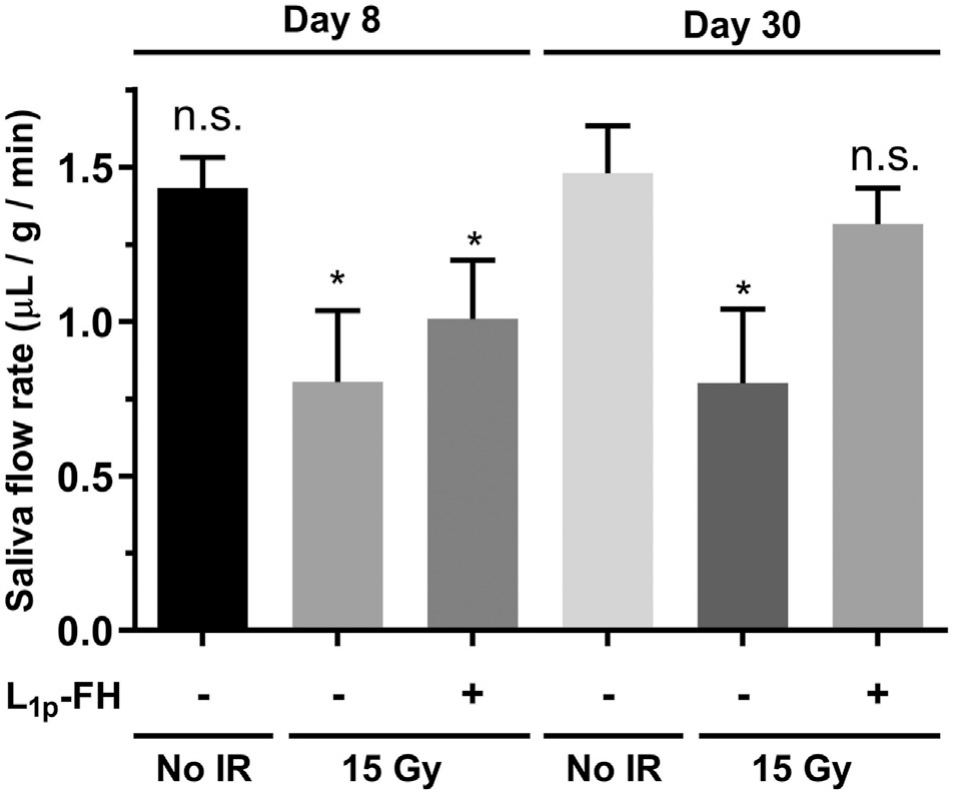
L_1p_-FH increases saliva secretion after radiation treatment. Mice were anesthetized and stimulated with pilocarpine and isoproterenol at days 8 and 30 with saliva collected for 5 min. Data represent the means ± SD of *n* = 5 mice per condition with statistical significance assessed using one-way ANOVA (*p < 0.01) and Dunnett’s post-hoc test for multiple comparisons to group 1 (non-irradiated mice at day 30). The symbol (+) indicates L_1p_-FH injection, while the symbol (−) indicates no L_1p_-FH injection, and n. s indicates no significant differences from group 1 (non-irradiated mice at day 30).

Our previous studies indicate that treatment with L_1p_-FH enhances saliva secretion in a wounded SMG mouse model (Nam et al., 2017a; Nam et al., 2017b; Nam et al., 2019b). To determine whether similar effects occur in an irradiated mouse model, we treated irradiated SMG with a transdermal injection of L_1p_-FH as described in Materials and Methods. As shown in Figure 5, group 1 (non-irradiated glands) showed intact saliva flow rates (i.e., 1.43 μL/g/min), as expected. In contrast, group 2 (irradiated untreated glands) exhibited a significant reduction in saliva flow rates (i.e., 0.80 μL/g/min, *n* = 5, *p* < 0.01). Notably, group 3 (irradiated glands treated with L_1p_-FH) showed a significant increase of saliva flow rates (1.32 μL/g/min, *n* = 5, *p* < 0.01) at day 30, thereby demonstrating that L_1p_-FH restores saliva secretion after radiation treatment.

## Discussion

Our previous studies indicated that treatment with FH alone promotes neither cell polarity nor differentiation in salivary gland epithelium, both *in vitro or in vivo* (Nam et al., 2016; Nam et al., 2017a; Nam et al., 2017b; Nam et al., 2019b; Dos Santos et al., 2021). However, specific L_1p_ sequences (A99: CGGALRGDN-amide, YIGSR: CGGADPGYIGSRGAA-amide) proved to be useful for improving salivary gland regeneration (Hoffman et al., 1998). Specifically, freshly isolated SMG cells grown on L_1p_ chemically attached to FH induced lumen formation and secretory function (Nam et al., 2016). Moreover, L_1p_-FH promoted salivary gland regeneration in an *in vivo* wound-healing mouse model (Nam et al., 2017a; Nam et al., 2017b), thus leading to increased saliva secretion. Such functional recovery indicates that FH-based scaffolds can be used to promote salivary gland function in radiation-induced hyposalivation. Additionally, we developed a transdermal delivery system specifically for this study with the aim of using the patient’s own blood for polymerization to increase biocompatibility (Froelich et al., 2010; Dietrich et al., 2013) and having the ancillary benefits of displaying optimal rheological properties (i.e., softness) and being less invasive than other delivery methods (i.e., retro-ductal delivery (Nair et al., 2016) and surgical application (Ogawa et al., 2013)), all of which indicates a greater degree of clinical applicability for our newly designed mouse model.

Regarding results of the current study, salivary gland morphology was significantly improved by L_1p_-FH (Figures 2I–L and Figure 3D,E) and saliva secretion (Figure 5) was likewise restored by day 30 post-radiation; however, such treatment gains cannot be counted on to persist, given the residual fibrosis noted (Figure 2L). Additionally, future studies will use growth factors specifically targeted for angiogenesis (i.e., VEGF and FGF9) (Nam et al., 2019b) in response to current results demonstrating L_1p_-FH promoted macrophage polarization (Figure 4) but gave rise to no blood vessel formation (Supplementary Figure S1). Moreover, should such gains in fact prove persistent (e.g., maintained over long periods of time), we as yet have limited knowledge of the mechanisms responsible for this recovery. These issues notwithstanding, the results to date are important because they are the first time that L_1p_-FH has been used in irradiated glands to restore their form and function.

It is noteworthy to mention three major differences between our previous studies and the current work. First, our previous studies used L_1p_ in trimeric form (Dos Santos et al., 2021) and in combination with growth factors (Nam et al., 2019b), while the current work employs only monomeric forms and no growth factors. Next, our previous studies used a more invasive SMG surgical punch model (Nam et al., 2017a; Nam et al., 2017b; Nam et al., 2019b) as compared to currently used transdermal injection implantation method. Finally, we replaced the SMG wounded mouse model of our prior studies with a radiation model for greater specificity in terms of clinical features and increased translational application.

To expand on this work, future studies will perform extended saliva secretion studies and track the appearance of fibrosis at multiple time points via histological studies and investigate how L_1p_ used here (i.e., A99 (Mochizuki, 2003; Rebustini et al., 2007; David et al.,2008) and YIGSR (Caiado and Dias, 2012; Frith et al., 2012; Huettner et al., 2018; Motta et al., 2019)) bind to specific integrins, thus addressing the questions noted above in relation to treatment duration and mechanisms. Finally, should this treatment near the stage of clinical trials, it would be important to replace the current single dose of radiation used for proof of concept and early exploration with more clinically appropriate fractionated doses.

## Data Availability

The original contributions presented in the study are included in the article/Supplementary Material, further inquiries can be directed to the corresponding author.

## Supplementary Material

Supplemental Figure S1 L_1p_-FH does not enhance angiogenesis after radiation treatment. VCAM-1 (green) and ICAM-1 (red) were analyzed using confocal microscopy. Scale bars represent 100 µm. Representative image from a total of 5 mice per group.
