# Supplementary figures and images for: Laminin-1 Peptides Conjugated to Fibrin Hydrogels Promote Salivary Gland Regeneration in Irradiated Mouse Submandibular Glands
Source: Front Bioeng Biotechnol. 2021 Sep 24;9:729180. doi: 10.3389/fbioe.2021.729180 (PMC8498954; doi:10.3389/fbioe.2021.729180)

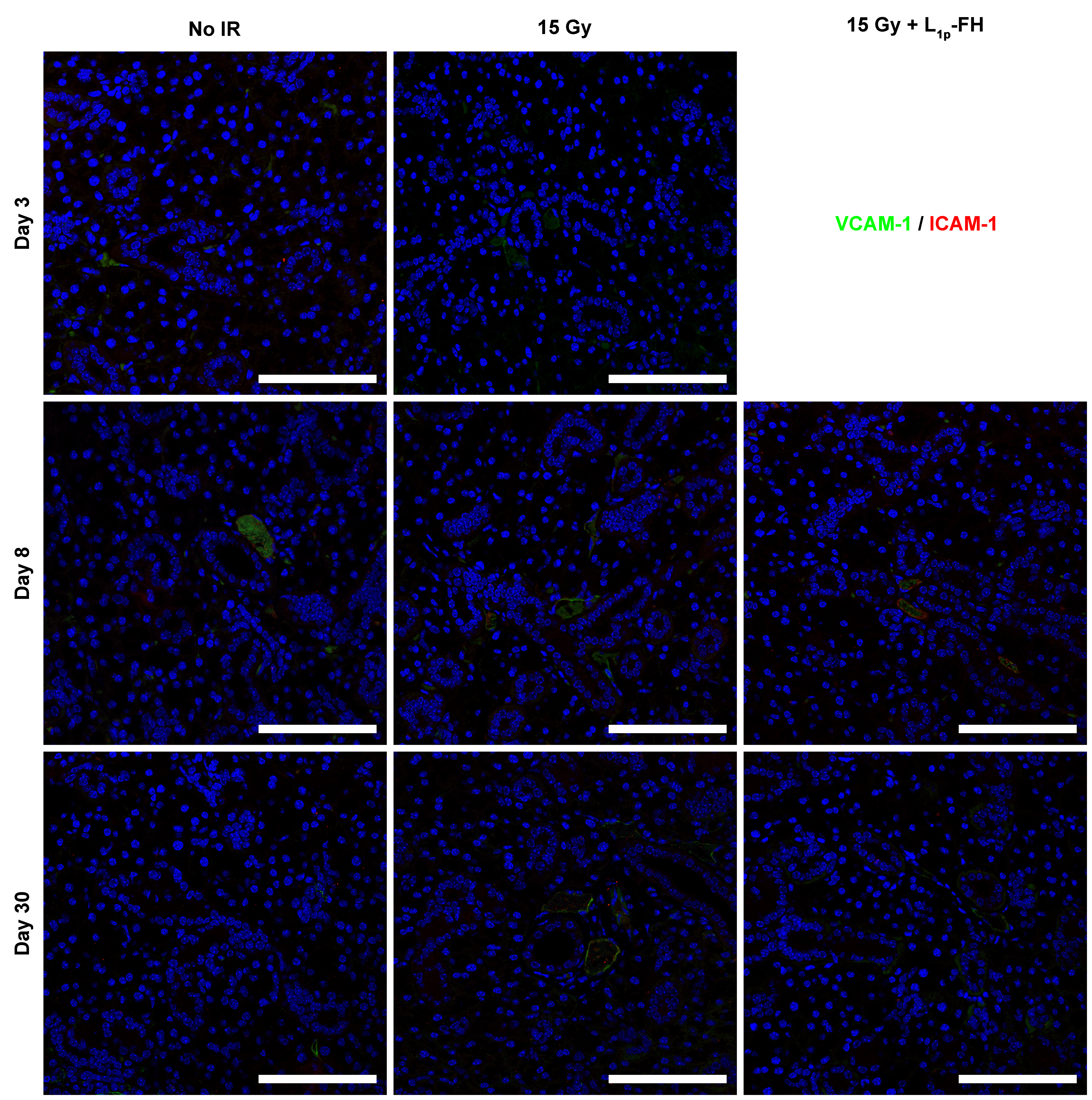

Supplement: Supplementary file 1 [file Image1.JPEG]
